# American Association of Obstetricians and Gynecologists

**Published:** 1906-11

**Authors:** Adam H. Wright


### American Association of Obstetricians and Gynecologists.

[*Editorial in Canadian Practitioner and Review, October.*]

The Nineteenth Annual Meeting of the American Association of Obstetricians and Gynecologists was held at the Hotel Havlin, Cincinnati, September 20, 21 and 22, 1906.

There are certain incidents in connection with the organisation and subsequent history of this admirable association which are sufficiently interesting to be worthy of regard at the present time. In the year 1886 it was decided to form a Congress of representative physicians and surgeons of the United States. Preliminary invitations were sent to the various special societies asking for co-operation. The American Gynecological Society was the only one which refused to co-operate. In the official report of the Society for 1887 we find the following words: "The proposition to become part of the American Congress of Physicians and Surgeons was not adopted." The promoters of the proposed confederation were naturally disappointed because they desired a representation of the important subjects of Obstetrics and Gynecology. As a consequence of this disappointment there was a conference of some friends of the Congress. As the result of such conference the American Association of Obstetricians and Gynecologists was organised, not in opposition to any other society, but rather in the interest of the new Congress. After the organisation was fairly completed a formal application for admission was sent to the Congress. In the meantime, however, a change had come over the society which had formerly opposed the proposed Congress. We were not told whether this marvellous change was brought about in consequence of the organisation of the new society; but it was certainly a singular coincidence that the applications from the old Gynecological Society and the new Association of Obstetricians and Gynecologists for admission to the Congress were practically made at the same time. After some deliberation by the executive authorities of the Congress it was decided that the society which had shown pronounced hostility to the Congress up to the time of its sudden conversion should be received, and the new organisation which had been formed to assist the confederation in a serious emergency should be put on trial for a couple of years. In accordance with this remarkable decision the following resolution was passed: "Resolved that it is the sense of this Executive Committee that they will not consider the application of any society which has not held at least two annual meetings."

The new association entered into its period of probation with feelings of both surprise and disappointment, but with hopes that its work would be judged on its merits, and duly recognised at the proper time. After the second annual meeting the volumes of the transactions for two years were duly filed. After the third meeting, which was held in Philadelphia, twelve copies of each volume of transactions were asked for. This meeting was so successful from every point of view that it was supposed the perusal of the transactions would strengthen any favorable impressions which had been created by the former two. The delay was to some extent embarrassing because the association was unable to announce definitely the time and place of the fourth meeting. The third volume was completed as soon as possible, and the thirty-six books were forwarded to the committee of the Congress. When all the evidence was received the committee did not arrive at a conclusion suddenly or rashly; they took ample time for deliberation, and while they were deliberating the new Association was waiting. After about seven months the Executive Committee of the Congress of American Physicians and Surgeons held a meeting in Philadelphia and shortly afterward sent an official intimation to the new society, stating that it would not be admitted to the Congress.

This is a brief but plain statement of one of the most extraordinary transactions known to medical history in North America. Why was the new association accorded such treatment? This question was asked by the president of this association fifteen years ago, and has not yet been answered. There was at that time a rumor in the air, that the general argument used against the new association was that it really represented nothing more than a duplication of the work of other sections, and for that reason should not be admitted.

This statement of the position is taken from the address of the president of 1891. With reference to the "duplication of the work of other sections," the president spoke as follows: "I have nothing to do with such an argument, and care not whether it be considered good, bad or indifferent. I shall remove the necessity of using it by saying that we concede that the Congress had a perfect right to refuse to admit us if its members thought fit. We insist, however, that it had no right to subject us to humiliation such as this. It had no right to place us on probation for an extended period, and then absolutely ignore the essence of the implied contract between them and us. The resolution of the Congress required certain things from us. We have fulfilled those requirements in every part. We actually came into existence in the interests of the Congress. We have supported it loyally in every particular. We have shown no particle of antagonism to any of its sections; we have patiently submitted to much inconvenience through the delay in sending its singular ultimatum. Is it possible that the majority of the members of that great organisation will feel proud of the actions of their Executive? I have considered the matter in all its aspects, and I cannot conceive how the members of the Congress can reasonably defend the methods of their committee."

The president then spoke as follows respecting the future prospects of the association: "Well, gentlemen, what are we to do now? It gives me unbounded pleasure to assure you that our Executive Council holds no divided opinions. The necessities of the case compel us to bid the Congress a sad farewell, but in doing so we indulge in the hope that we may be permitted to continue our existence, which we have found exceedingly pleasant, as well as extremely profitable. Our association is alive today, it is going to live, it is going to thrive, it is going to do a great work on this vast continent. I say this in no boasting spirit. I desire to assume no air of bravado. I feel fully impressed with the responsibility I assume when I say that we have a grand future before us. I, who have done so little for you, can express myself with greater freedom than can others who have borne so nobly the burden of organising this magnificent society. I have witnessed the efforts of our founders with profound admiration. I have watched their zeal, their devotion, their untiring energy, with a feeling of wonder. I have viewed their boundless enthusiasm, their wondrous capacities for work, and their unselfish devotion to each other and our common cause with perfect delight. In addition it gives me great pleasure to refer to the dignified bearing of our councillors under somewhat trying circumstances. I know of no act on our part that will ever bring a blush of shame to any of our members. It appears to me that our prospects were never brighter than they are today. The object of our association, "the cultivation and promotion of knowledge in whatever relates to abdominal surgery, obstetrics and gynecology," is ever kept in view by one and all, and the results in three short years, the evidence of which may be found in the three volumes of our transactions, will inspire us with confidence and fill us with hope in the future.

"Let it be our duty as well as our pleasure to worthily continue the work which has been so auspiciously begun. Let envy, hatred and all uncharitableness toward other societies be ever kept far from us. Let us forget the indignities which have been heaped upon us. Let our memories of the past pertaining to our own work ever remain as pleasant as they are to-day."

Has the society realised the somewhat lofty expectations thus expressed? It would seem not unprofitable to consider some of the features of the meeting held at Cincinnati. One noticed very soon that the "old guard," the founders, are still on deck and ever ready for action. Among the founders present were Potter, Price, Carstens, Ill, McMurtry, Reed, Taylor, Miller and Werder. The able and active secretary has a genius for organisation and executive work, and looks quite as young as he did fifteen years ago. McMurtry, Reed, Price, and Carstens show no signs of getting old, they are as vigorous, keen and alert in debate as ever. Among others present were a number who joined shortly after the organisation of the society and are still active members, such as Ross, Hall, Longyear, Murphy, Morris, Ricketts, Zinke, Hayd.

We learn that others of the founders and members had expected to be present, but were prevented by unforseen circumstances. There is also much new and young blood. To one attending the session of the first forenoon there appeared in the chair a boyish looking fellow with a clean-cut man's face. Who is that? He is the president, John Young Brown, a Kentucky boy, born in Louisville, a son of one of Kentucky's ablest Governors, now one of the leading surgeons of St. Louis. After a time a well groomed boy with an intelligent man's face takes the chair. Who is that? He is another Kentucky boy, now in New York, one of the vice-presidents. These men presided with a grace and dignity that was altogether admirable. The association has shown much wisdom in holding out the glad hand to bright young surgeons and obstetricians, and bringing them into the fold.

The meeting lasted three days of two sessions each, morning and afternoon. The attendance was large, the room being filled most of the time. It is impossible to give a condensed report in the space at our disposal which would give any adequate conception of the character of the papers and the discussions. Some of the papers were far above the average, and some of the discussions were extremely good, and were listened to with breathless interest by those present. There was a general consensus of opinion that the meeting was one of the best that the association has ever known. One may go, perhaps, a little further, and say, without any fear of contradiction on the part of those in attendance, that it was one of the best medical meetings ever held in North America.

One cannot speak too highly of the work done by the Committee of Arrangements, of which Dr. Bonifield was chairman and Dr. Tate was secretary. One can also positively state that the unbounded and generous hospitality of the resident fellows will ever be remembered by those who had the privilege of attending this great meeting in Cincinnati.

In conclusion I may answer the question as to "lofty expectations" by saying that, although I feared that the enthusiasm of the president of fifteen years ago was causing him to soar a little too high, I now think, after attending this meeting, that the results have fully justified his statement: 'The association is alive to-day, it is going to live, it is going to thrive, it is going to do great work on this continent."

ADAM H. WRIGHT.

